# Thymidine phosphorylase expression is associated with time to progression in patients with metastatic colorectal cancer

**DOI:** 10.1186/1472-6890-14-25

**Published:** 2014-06-10

**Authors:** Elinor Bexe Lindskog, Kristoffer Derwinger, Bengt Gustavsson, Peter Falk, Yvonne Wettergren

**Affiliations:** 1 Department of Surgery, Institute of Clinical Sciences, Sahlgrenska Academy, University of Gothenburg, Gothenburg 416 85, Sweden

**Keywords:** Colorectal neoplasms, PD-ECGF, Chemotherapy, Serum, Biological markers

## Abstract

**Background:**

5-Fluorouracil (5-FU) is the cornerstone of chemotherapeutic treatment for patients with colorectal cancer. The enzyme thymidine phosphorylase (TP) catalyzes the conversion of 5-FU to its active metabolite, 5-fluoro-2’-deoxyuridine. TP is expressed in tumour epithelial cells and stromal cells, particularly in tumour-associated macrophages. These macrophages may affect sensitivity to chemotherapy. Previously, we identified TP as a predictive factor in microdissected tumour samples of patients with advanced colorectal cancer. In the present study, we analysed *TP* expression in tissues and associated stromal cells from patients with advanced colorectal cancer and associated *TP* levels to tumour response and time-to-event variables during first-line chemotherapy treatment. We also investigated the association between serum TP levels at the time of surgery and gene expression in primary tumour tissues.

**Methods:**

This study included 125 patients with metastatic colorectal cancer treated with first-line 5-FU-based chemotherapy. To quantify *TP* gene expression levels in tumour tissues, real-time polymerase chain reaction was performed using the 7500 Fast Real-Time PCR system (Applied Biosystems, Foster City, CA, USA). TP protein concentration in matched serum samples was determined using an enzyme-linked immunosorbent assay system (USCN Life Science Inc.).

**Results:**

The tumour response rate was 31%, and 30% of patients exhibited stable disease. No associations between *TP* expression level and age or gender were observed. Levels of *TP* mRNA in mucosa and tumours were positively correlated (r = 0.41, p < 0.01). No correlation between *TP* expression and tumour response rate was observed. Time to progression was significantly longer in patients with high *TP* expression (p < 0.01). Serum TP protein levels were not associated with tumour response or time-to-event variables and did not correlate with gene expression in tumour tissues.

**Conclusions:**

High *TP* gene expression in non-microdissected tumour tissues of patients with advanced colorectal cancer correlates with longer time to progression, which could be related to treatment. These results are in contrast to previous studies where microdissected tumour cells were analysed and may be due to the presence of adjacent stromal cells. Serum TP protein expression does not correlate to *TP* gene expression in tissues of patients with advanced colorectal cancer.

## Background

To date, 5-fluorouracil (5-FU) constitutes the fundamental basis of chemotherapy treatment for patients with colorectal cancer. The enzyme thymidine phosphorylase (TP; E.C. 2.4.2.4) catalyzes the conversion of 5-FU to its more active nucleoside form, 5-fluoro-2’-deoxyuridine, representing one of the main pathways by which this drug exerts its cytotoxic effect [1]. In the cell, TP is involved in pyrimidine metabolism. Previous studies have shown that TP levels are higher in tumour compared with normal tissues in a wide range of solid tumours [2-5]. TP and its catalytic product, 2-deoxy-D-ribose-1-phosphate act as angiogenic factors via induction of endothelial cell migration and tube formation [6-9]. In hypoxic environments, TP may also impart resistance to apoptosis [1,10]. TP expression is observed in tumour epithelial cells and stromal cells, particularly in tumour-associated macrophages (TAMs) [4,8,11].

Previously, we identified *TP* gene expression in microdissected tumour samples from colorectal carcinomas as a possible predictor of chemotherapy response and survival in advanced colorectal cancer [12]. This finding was in keeping with the observations of other independent reports [12-15]. In the present study, we included tumour-associated stromal cells in our analysis of *TP* gene expression, because the majority of expression appears to be associated with TAMs. These specific macrophages play an important role in the tumour microenvironment and may affect sensitivity to chemotherapy [16].

Previous studies have shown that plasma TP is elevated in cancer patients [17,18]. Furthermore, high serum TP (sTP) in patients with oesophageal and uterine cervical cancer appears to be related to poor prognosis and inferior response to chemotherapy [18,19]. In patients with colorectal cancer, high levels of sTP in venous blood drainage specimens were positively correlated with tumour stage, poor prognosis and particularly, with risk of liver metastasis [20]. However, to the best of our knowledge, no studies investigating TP levels in peripheral blood samples of colorectal cancer patients have been conducted. The primary aim of this study was to investigate the relationship between tissue levels of TP in patients with advanced colorectal cancer and tumour response and time-to-event variables during first-line chemotherapy treatment. Our second aim was to examine whether *TP* gene expression in tumour tissues reflects TP protein expression in serum samples at the time of surgery.

## Methods

### Patients and study design

This retrospective study included 125 patients with metastatic colorectal cancer, treated with first line 5-FU-based chemotherapy. Forty-one patients had previously received adjuvant chemotherapy. Fifty-six patients had rectal cancer and 61% of them received pre-operative irradiation. All patients were treated at Sahlgrenska University Hospital (Östra, Gothenburg, Sweden) between 2002 and 2011, and patients were followed up with CT scans every 3 months during treatment. Treatment response was evaluated according to criteria outlined by the World Health Organization [21]. As first-line chemotherapy, 111 patients were treated according to the Nordic FLV-protocol (500 mg/m^2^ of 5-FU in combination with 60 mg/m^2^ of leucovorin, given as a single treatment (n = 31) or in combination with 85 mg/m^2^ oxaliplatin (n = 53) or 180 mg/m^2^ irinotecan (n = 27). Fourteen patients received the 5-FU pro-drug capecitabine as a single treatment (n = 4), or in combination with oxaliplatin (n = 3) or irinotecan (n = 7). First-line therapy was continued until evidence of disease progression occurred, unacceptable toxicity developed, or the patient elected to withdraw. Palliative survival was defined as the time from first-line treatment until the date of death. Ninety-nine patients died during the follow-up period, while 26 remained alive [these patients were censored in the survival analysis, with a median follow-up time of 871 days (range 331–2766)]. Radiological time to tumour progression was defined as the time from initiation of first-line treatment to tumour progression, and 40 patients were censored for time to progression data. This study was approved by the Regional Ethical Review Board in Gothenburg (EPN, Ö445-00).

### Tissue and serum sampling

Tumour samples (n = 125) and matched macroscopically normal-appearing mucosa (obtained approximately 10 cm from the tumour, n = 125) were obtained from patients at the time of primary surgery, snap-frozen in liquid nitrogen and stored at −80°C until analysis. Matched venous blood samples, which were collected pre-operatively in a standardized manner, were available from 70 of the 125 patients. For serum sampling, one tube without anticoagulant was left at 20°C for 30 min. Samples were centrifuged for 10 min at 1519 × g and serum was gently collected and frozen in small aliquots for downstream assays.

### Total RNA extraction, cDNA preparation and real-time quantitative polymerase chain reaction (PCR)

Total RNA was isolated from 10 to 30 mg tissue using the High Pure RNA Tissue Kit (Roche Diagnostics GmbH). cDNA was synthesized using the High Capacity cDNA Reverse Transcription Kit (Applied Biosystems, Foster City, CA, USA). Real-time quantitative PCR was performed using the 7500 Fast Real-Time PCR system (Applied Biosystems). *TP* transcript levels were quantified using Assays-on-Demand™ from Applied Biosystems (Hs00157317_m1). β*-actin* was used as an endogenous control to normalize for RNA levels and efficiency of the reverse-transcription reaction. Primer and probe sequences (Table 1) and multiplex PCR conditions were previously described [22].

**Table 1:** Primer and probe sequences used in the real-time quantitative polymerase chain reaction

**TP**	**Probe**	**5′-CAG CCA GAG ATG TGA CAG CCA CCG T-3′**
	forward primer	5′-CCT GCG GAC GGA ATC CTA TA-3′
	reverse primer	5′-TGT GAT GAG TGG CAG GCT GT-3′
**β-actin**	probe	5′-CCT GAA CCC CAA GGC CAA CCG-3′
	forward primer	5′-CGT GCT GCT GAC CGA GG-3′
	reverse primer	5′-GAA GGT CTC AAA CAT GAT CTG GGT-3′

Abbreviations: *TP* Thymidine Phosphorylase.

### Enzyme-linked immunosorbent assay (ELISA)

The concentration of TP protein in patient serum samples (sTP) was determined using a commercially available ELISA system (E90948Hu; USCN Life Science Inc., Wuhan, China) according to manufacturer’s instructions for quantitative measurements. The minimum detection limit was < 0.25 ng/mL and the intra- *vs* inter-assay variation (CV%) was < 10% and 12%, respectively, as stated by the manufacturer. Absorbance was measured at 450 nm using a plate reader and serum protein concentration was calculated using associated software (V-max/Softmax Pro; Molecular Devices, USA). Results were normalized to total protein content using a 96-well based assay method from Bio-Rad (Hercules, CA, USA) according to the method described by Lowry *et al*. [23].

### Statistical methods

Data were analysed by statistical modelling using JMP commercial software (version 10; SAS Inc., Cary, NC, USA). Differences between groups were calculated using the Kruskal–Wallis’ test or the Pearson’s Chi-square test. The Pearson correlation coefficient (r) was used to compare sets of continuous parameters measured in the same tissue. The relative real-time quantitative PCR values and protein levels of TP were dichotomized for subsequent time-to-event analysis with respect to the median value of expression. Tumours with gene expression levels lower or higher than the median value were classified as low or high expression status, respectively. Survival and time-to-event curves were calculated by the Kaplan–Meier method and statistically significant differences in survival were calculated using the log-rank test. Relative risk was assessed by uni- and multivariate Cox proportional hazard model. Statistical values of p ≤ 0.05 were considered significant.

## Results

### Relative TP gene expression

Patient and tumour characteristics are presented in Table 2. The median age of all patients (n = 125) was 65 years and 46% of patients were female. There was no association between relative tumour *TP* (*tTP)* gene expression and age or gender. The expression of *tTP* was associated with tumour location, with rectal carcinomas exhibiting higher median *tTP* expression compared with colon carcinomas (0.32, IQR 0.10–0.57 and 0.15, IQR 0.060–0.37, respectively, p < 0.01). This difference in *TP* gene expression was not observed in mucosa samples. We also observed a positive correlation between *tTP* and mucosa *TP* (*mTP,* r = 0.41, p < 0.01).

**Table 2:** Patient and tumour characteristics subgrouped according to high or low thymidine phosphorylase expression

	**Patient cohort**		Patient subgroup for serum analysis*	
	**(n = 125)**		**(n = 70)**	
	***tTP *** **high**	***tTP *** **low**	**sTP high**	**sTP low**
**Age (year)**				
Median (IQR)	68 (59–75)	64 (57–73)	66 (56–75)	65 (59–74)
**Gender (%)**				
Female	27 (44)	31 (49)	15 (43)	12 (34)
Male	35 (56)	32 (51)	20 (57)	23 (66)
**Tumour location (%)**				
Colon	26 (42)	43 (68)	21 (60)	18 (51)
Rectum	36 (58)	20 (32)	14 (40)	17 (49)
**Tumour stage (%)**				
I	4 (6)	-	1 (3)	-
II	17 (27)	4 (6)	6 (17)	6 (17)
III	24 (39)	27 (43)	12 (34)	16 (46)
IV	17 (27)	32 (51)	16 (46)	13 (37)
**Tumour differentiation (%)**				
Well	1 (2)	1 (2)	-	1 (3)
Medium	42 (68)	36 (57)	23 (66)	23 (66)
Poor	16 (26)	20 (32)	9 (26)	8 (23)
Mucinous	2 (3)	6 (10)	3 (9)	3 (8)
Unknown	1 (2)	-	-	-
**Assessed lymph nodes, median (IQR)**	17 (12–24)	19 (16–25)	17 (14–26)	21 (14–29)
**Positive lymph nodes, median (IQR)**	1 (0–6)	4 (2–8)	3 (0–8)	3 (1–9)
**T-stage (%)**				
1	-	-	-	-
2	8 (13)	-	2 (6)	2 (6)
3	36 (58)	32 (51)	16 (46)	19 (54)
4	13 (21)	25 (40)	15 (43)	14 (40)
Not evaluable	5 (8)	6 (10)	2 (6)	-
**N-stage (%)†‡**				
0	25 (40)	6 (10)	9 (26)	8 (23)
1a	8 (13)	9 (14)	6 (17)	5 (14)
1b	6 (10)	11 (17)	2 (6)	5 (14)
2a	9 (14)	15 (24)	7 (20)	5 (14)
2b	13 (21)	22 (35)	10 (28)	12 (34)
X	1 (2)	-	1 (3)	-
**Metastatic sites (%)**				
Liver	24 (39)	27 (43)	13 (37)	19 (549
Pulmonary	3 (5)	3 (5)	1 (3)	2 (6)
Skeletal	-	1 (2)	-	-
Local	3 (5)	2 (3)	3 (9)	-
Multiple including liver	19 (31)	17 (27)	13 (37)	5 (14)
Multiple excluding liver	8 (13)	10 (16)	4 (11)	4 (11)
Lymphatic	5 (8)	3 (5)	1 (3)	5 (14)

Sum of percentages may not add up to 100 due to rounding.

Abbreviations: *tTP* tumour thymidine phosphorylase, *sTP* serum thymidine phosphorylase, *IQR* interquartile range.

*Serum was obtained from 70 patients.

†TNM, 7th edition from UICC/AJCC (Union for International Cancer Control/American Joint Committee on Cancer).

‡Data missing on one patient due to palliative surgery.

**Figure 1:**
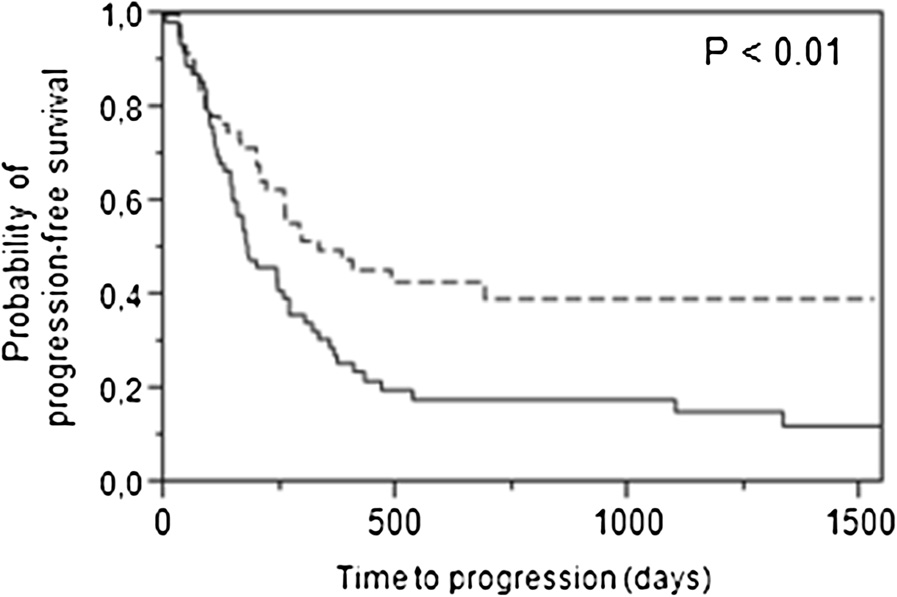
**Patients with high thymidine phosphorylase (*TP) *** **gene expression (n = 62, dotted line) in tumour tissues exhibited significantly longer time to progression compared with patients with low expression (n = 63, solid line).**

The total tumour response rate was 31% (complete response 4%, partial response 27%) and 30% of patients exhibited stable disease. Patients were grouped according to high/low *tTP* gene expression using a median value cut-off (0.20, IQR 0.080–0.51). While no relationship between tumour response during treatment and *tTP* expression was observed, we noted that time to progression was extended in the high *tTP* expression group (p < 0.01, Figure 1). Cox multivariate analysis showed that *tTP* gene expression was significantly associated with time to progression (Table 3), and independent of other parameters included in the model. No correlation between *tTP* expression and palliative survival was observed. All patients with stage I disease (4/4) and two-thirds of patients with stage II disease (14/21) were in the high *tTP* expression group. There was no association between *mTP* gene expression (median value 0.29, IQR 0.19–0.44) and tumour response or time-to-event variables.

**Table 3:** **Cox univariate and multivariate analyses demonstrating the influence of clinicopathological parameters and *TP *** **gene expression on time to progression**

	**Univariate**		**Multivariate**		
	**HR**	95% CI P*	**HR**	**95% CI**	P*
**Gender**					
Male	1		1		
Female	1.46	0.95-2.25 NS	1.48	0.94-2.34	NS
**Age (years)**	1.00	0.99-1.03 NS	1.01	0.98-1.04	NS
**Tumour location**					
Rectum	1		1		
Colon	1.49	0.96-2.34 NS	1.21	0.74-2.01	NS
**Tumour differentiation**					
Well/Medium	1		1		
Poor	1.65	1.03-2.61	1.50	0.92-2.42	
Mucinous	2.04	0.84-4.23 NS	2.12	0.83-4.78	NS
**TP gene expression**					
High	1		1		
Low	1.78	1.15-2.79 < 0.05	1.61	1.01-2.61	<0.05
**ECOG performance status**					
0	1		1		
1	1.09	0.62-1.81	1.18	0.67-1.98	
2-3	6.51	2.44-14.5 < 0.05	7.17	2.60-16.8	<0.05
**Chemotherapy**					
Combination treatment^†^	1		1		
Single treatment^‡^	1.29	0.78-2.07 NS	1.11	0.59-2.05	NS

Abbreviation: *HR* Hazard Ratio, *CI* Confidence interval, *TP* Thymidine Phosphorylase, *ECOG* Eastern Cooperative Oncology Group *Statistical method; Cox proportional hazards regression. ^†^5-fluorouracil or capecitabine in combination with oxaliplatin or irinotecan; ^‡^5-fluorouracil or capecitabine.

### TP serum protein levels

TP protein levels were analysed in serum samples of 70 patients. Patient demographic information and tumour characteristics of these patients were similar to those described for the whole study population (Table 2). There was no correlation between sTP levels, age, gender or tumour location. No significant correlation between sTP at the time of surgery and *tTP* or *mTP* was observed. Patients were sub-grouped according to high/low sTP protein expression using a median cut-off value [0.072 ng/mg (total protein content), IQR 0.044–0.11]. This cut-off point was used in time-to-event curves. No association between sTP and tumour response or time-to-event variables was observed.

## Discussion

High levels of TP in tumour tissues and/or serum may be indicative of poor prognosis, however, it may also be advantageous in the chemotherapeutic setting when 5-FU-based drugs are used [9,24-26]. In the present study, we assessed the expression of TP in patients receiving 5-FU-based chemotherapy as first-line treatment. We observed that high *tTP* gene expression was associated with longer time to progression, but not with tumour response. These results are in contrast to those observed in our previous study, which describes the expression of 18 5-FU-related genes in advanced colorectal cancer, where high *tTP* expression was associated with shorter time to progression and survival, and worse response. This discrepancy may be due to differences in the cell types being analysed. In our previous study, tumour tissue was microdissected, whereas the present study used both stromal and epithelial tumour cells. It is plausible that tumour stromal cells play an important role *in vivo*, during the evolution of primary cancer to metastatic disease. TP is predominantly expressed in the tumour stroma by macrophages [9,27] and TAMs play an important role in the tumour microenvironment [16,28].

Schwartz *et al.,* demonstrated that human colon carcinoma cells transfected with *TP* cDNA exhibited higher sensitivity to 5-FU compared with parental wild-type cells, and this phenomenon has also been shown in other cell lines [29-31]. Furthermore, in a recently published study of 76 colorectal cancer patients treated in the neoadjuvant setting, high *tTP* expression was associated with histopathological response [15]. In contrast, a study by Yanagisawa *et al.*, observed no association between *tTP* gene expression and clinical response in a cohort of 16 patients analysed in the palliative setting [32]. However, studies by Kumamoto *et al.,* and Metzger *et al.,* identified an association between low *tTP* gene expression and response when 45 primary colorectal carcinomas and 38 metastatic lesions were analysed, respectively [13,14]. In the latter three studies, the tissue samples were microdissected and analysis of *TP* gene expression was thus performed predominantly on epithelial tumour cells. Other factors contributing to discrepant results between different studies may be related to the composition of the patient cohort with regard to demographic and clinicopathological parameters. For instance, patients with stable disease were included in the present, but not in our previous study.

Whether gene and protein expression actually reflect enzyme activity, is a major question in these studies. This issue was addressed by Mimori *et al.,* who showed that *TP* mRNA detected by *in situ* reverse-transcriptase PCR was comparable with TP enzyme activity detected by high-performance liquid chromatography and enzyme assays [11]. It is also important to determine whether *TP* gene expression in the primary tumour is similar to expression in the evolved, metastatic lesion. Several studies indicate a positive correlation between *TP* expression in primary tumours and both synchronous and metachronous liver metastases, supporting the use of TP as a predictive factor for 5-FU-based chemotherapy in the palliative setting [13,33,34].

In a previous study of stage III colorectal cancer patients, we observed a positive correlation between *tTP* gene expression and numbers of positive lymph nodes, as well as higher *tTP* levels in tumours with worse differentiation grades [25]. In the present study, *tTP* expression was high in all stage I patients and in two-thirds of stage II patients. This result implies that patients with high *tTP* expression in primary tumours may have a worse prognosis, regardless of tumour stage.

In the present study, we analysed sTP obtained at the time of surgery as a possible surrogate marker for *TP* gene expression in primary tumours of patients with advanced CRC. sTP protein expression did not correlate with gene expression in matched tumour tissue. To evaluate the potential use of sTP as a predictive factor, measurement of sTP before and during treatment in a large patient cohort is therefore necessary.

## Conclusions

We demonstrate that high *tTP* gene expression in non-microdissected tumour tissue of patients with advanced colorectal cancer correlates with longer time to progression, which is related to the effect of treatment. This result is in contrast to those of previous studies where microdissected epithelial tumour cells have been analysed and may reflect the presence of stromal cells in the analysed tissue. The results of the present study suggest that sTP protein expression is not a useful surrogate marker for *TP* gene expression in primary tumour tissue of patients with advanced CRC.

## Abbreviations

5-FU: 5-Fluorouracil; TP: Thymidine phosphorylase; TAM: Tumour-associated macrophages; sTP: Serum TP; *tTP*: Tumour *TP*; *mTP*: Mucosa *TP.*

## Competing interests

The authors declare that they have no competing interests.

## Authors’ contributions

Conceived and designed the experiments: YW, EBL, PF, BG. Analysed the data: EBL, YW, KD, PF, BG. Wrote the manuscript: YW, EBL. All authors read and approved the final manuscript.

## Pre-publication history

The pre-publication history for this paper can be accessed here:

http://www.biomedcentral.com/1472-6890/14/25/prepub
